# Recent advances in N-heterocyclic carbene-based radical catalysis

**DOI:** 10.1039/d0sc01538e

**Published:** 2020-05-11

**Authors:** Takuya Ishii, Kazunori Nagao, Hirohisa Ohmiya

**Affiliations:** Division of Pharmaceutical Sciences, Graduate School of Medical Sciences, Kanazawa University Kakuma-machi Kanazawa 920-1192 Japan ohmiya@p.kanazawa-u.ac.jp; JST, PRESTO 4-1-8 Honcho, Kawaguchi Saitama 332-0012 Japan

## Abstract

In nature, a number of enzymes use thiamine diphosphate as a coenzyme to catalyze the pyruvate decarboxylation. The resultant enamine, a so-called “Breslow intermediate,” is known to perform single electron transfer to various electron acceptors. Inspired by this enzymatic catalysis, N-heterocyclic carbene (NHC)-catalyzed radical reactions have been developed. This minireview highlights the recent progress and developments in NHC-based radical catalysis. This minireview is categorized according to the reaction types; oxidation type reaction and carbon–carbon bond formation through single electron transfer/radical–radical coupling.

## Abstract 2

This minireview examines the history and state-of-the-art of the N-heterocyclic carbene-based radical catalysis.

## Graphical abstract

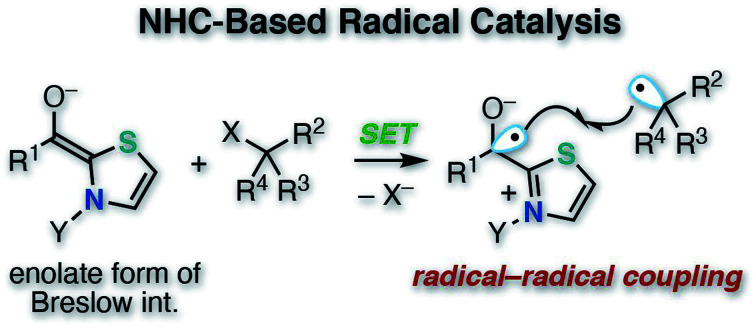

## 1. Introduction

**Scheme 1:**
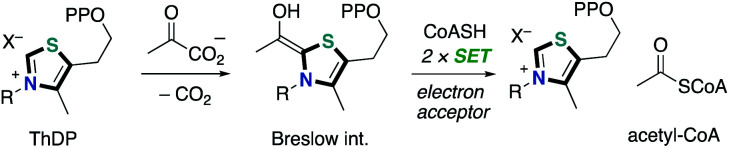
Enzymatic transformation of pyruvate.

**Scheme 2:**
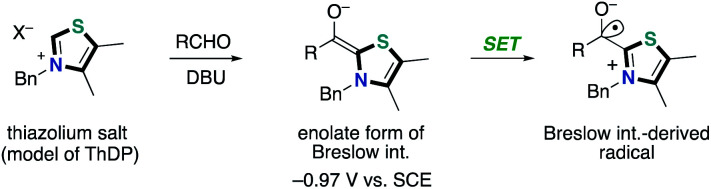
Redox properties of enolate form of Breslow intermediate.

N-heterocyclic carbene (NHC) catalysis, which exhibits a characteristic property to harness umpolung reactivity, has received considerable attention as a powerful tool for organic synthetic reactions. NHC catalysis through a two-electron reaction pathway has been extensively studied.^1^ The two-electron reaction process can access umpolung reactivity of carbonyls as acyl anions, enolates and homoenolates. On the other hand, NHC-catalyzed radical reactions through a one-electron reaction pathway are also known although the process remains challenging and much less developed. In biological systems, there are a number of enzymes utilizing thiamine diphosphate (ThDP, vitamin B1 derivative) as a coenzyme to catalyze the oxidative decarboxylation of pyruvate (Scheme 1).^2^ The resultant enamine, a so-called “Breslow intermediate”,^3^ performs single electron transfer to various electron acceptors such as lipoamides, flavin adenine dinucleotide and Fe_4_S_4_.^4^ The NHC-catalyzed radical reactions in the single-electron transfer (SET) manifold have been introduced in 2001.^5^ The crystal structure of the free radical intermediate of pyruvate ferredoxin oxidoreductase was disclosed. The redox and electron transfer properties of Breslow intermediates have been also studied by Fukuzumi and co-worker in late 1990s.^6^ They noted that a series of enolate form of Breslow intermediate derived from a thiamine analogue and aldehyde in the presence of excess amount of base, which has been alternatively called as active aldehyde, has extremely low oxidation potential (*E*_ox_ = −0.97 to −0.78 V *vs.* SCE) and small reorganization energy (*λ* = 12.0 to 12.9 kcal mol^−1^) (Scheme 2). These features make it a strong reducing agent. Additionally, the resultant Breslow intermediate-derived radical obtained by single electron oxidation was a persistent one.

Inspired by the enzymatic catalysis and these early studies, NHC-catalyzed radical reactions in organic synthesis have been developed to date. Due to the nature of highly reactive radicals, these reactions enabled the introduction of sterically bulky substituents, which used to be difficult in the well-known NHC catalysis involving a two-electron reaction pathway. This minireview highlights the recent progress and developments in the NHC-based radical catalysis. This minireview is categorized according to the reaction types; oxidation type reaction (Chapter 2) and carbon–carbon bond formation through SET/radical–radical coupling (Chapter 3).

## 2. Oxidation type reaction

**Scheme 3:**
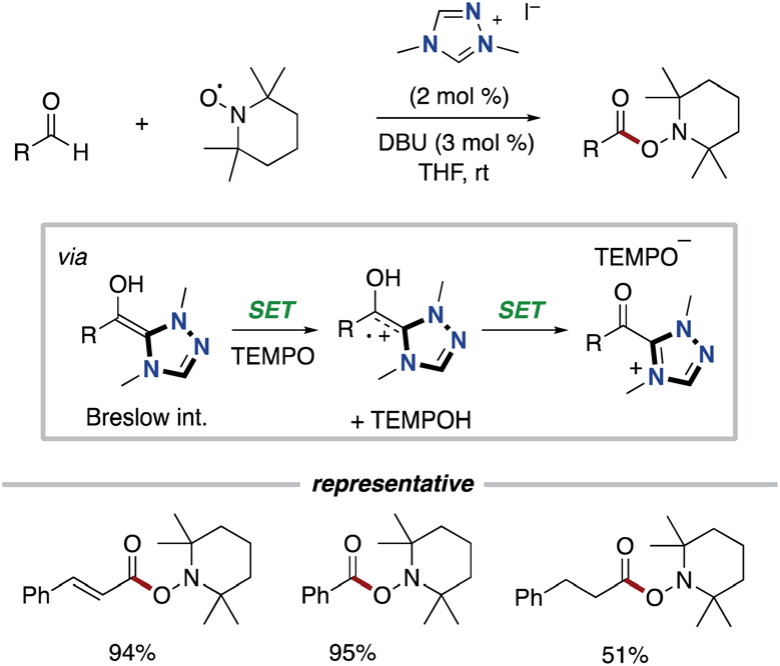
NHC-catalyzed oxidation of aldehydes to esters.

**Scheme 4:**
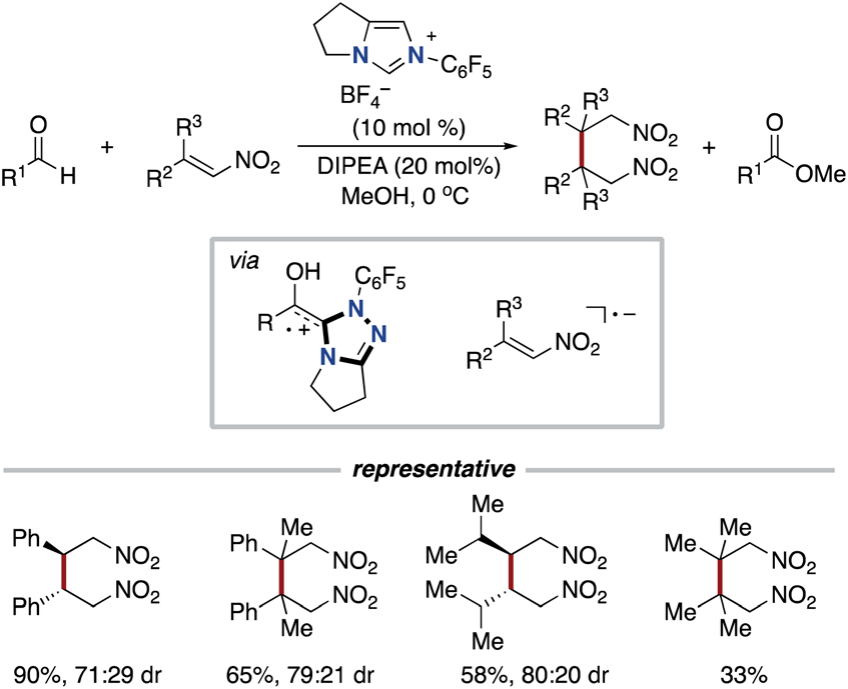
NHC-catalyzed reductive homo-coupling of nitroethylenes.

In 2008, Studer and co-workers reported the pioneering example of the synthetic reaction through NHC-based radical catalysis (Scheme 3).^7*a*^ The protocol enabled the oxidation of aldehydes to esters. This process involves two continuous single electron oxidations of Breslow intermediate by TEMPO to form the corresponding azolium ketone. In 2010, the Studer group used 3,3′,5,5′-tetra-*tert*-butyldiphenoquinone as the stoichiometric oxidant instead of TEMPO.^7*b*–*d*^ In 2014, Chi and co-workers reported the NHC-based radical catalysis for reductive homo-coupling reaction of nitroethylenes (Scheme 4).^8^ The reaction goes through nitroalkene-derived radical anions under SET process. In this process, the Breslow intermediate did not participate in the bond formation, which just acted as an electron donor.

**Scheme 5:**
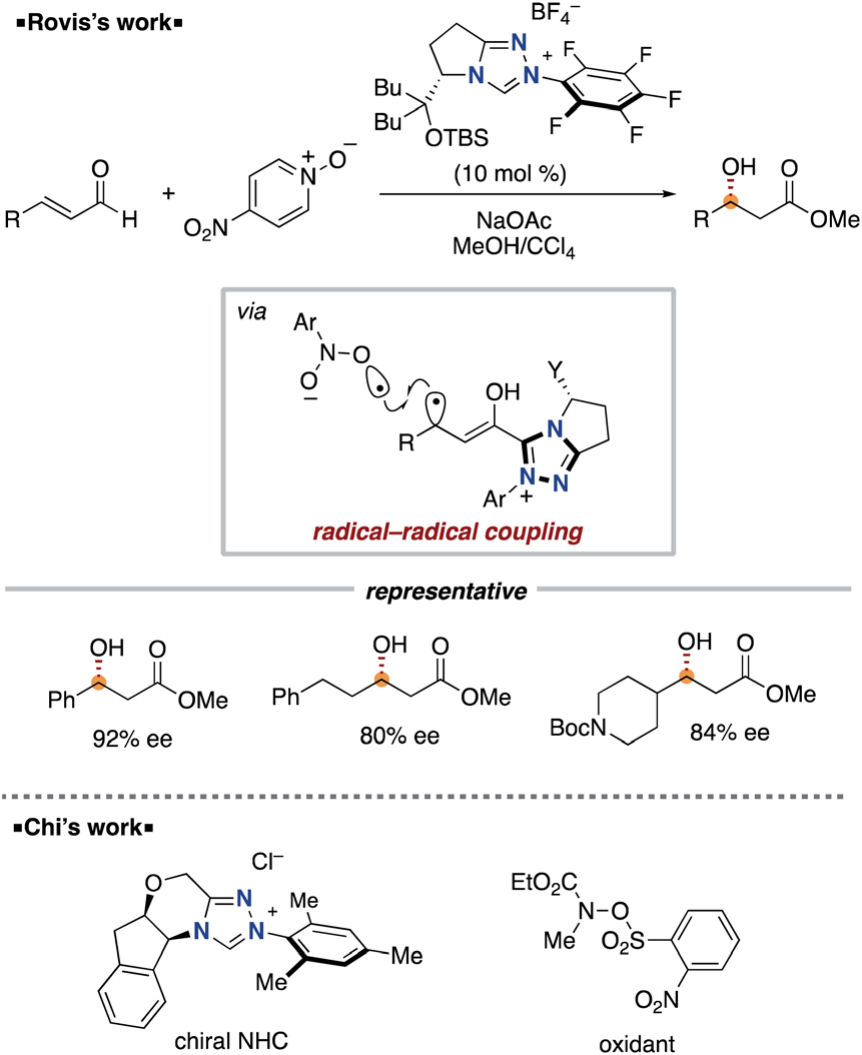
NHC-catalyzed enantioselective β-hydroxylatioin of enals.

The Rovis group^9^ in 2014 and the Chi group^10^ in 2015 independently reported asymmetric β-hydroxylation reactions of enals using chiral triazolium NHC catalyst (Scheme 5). This process involves the SET event between Breslow intermediate and nitrobenzene or the derivative followed by radical recombination between the resultant two radicals, a homoenolate-centered radical and an oxygen-centered radical. Based on this system, several radical β-functionalizations using enals have been developed.^11^ In 2015, the Rovis group reported the enantioselective synthesis of 3,4-disubstituted cyclopentanones through dimerization of enals.^12^ In 2017, the Ye group reported oxidative [3 + 2] annulation of dioxindoles and enals.^13^

**Scheme 6:**
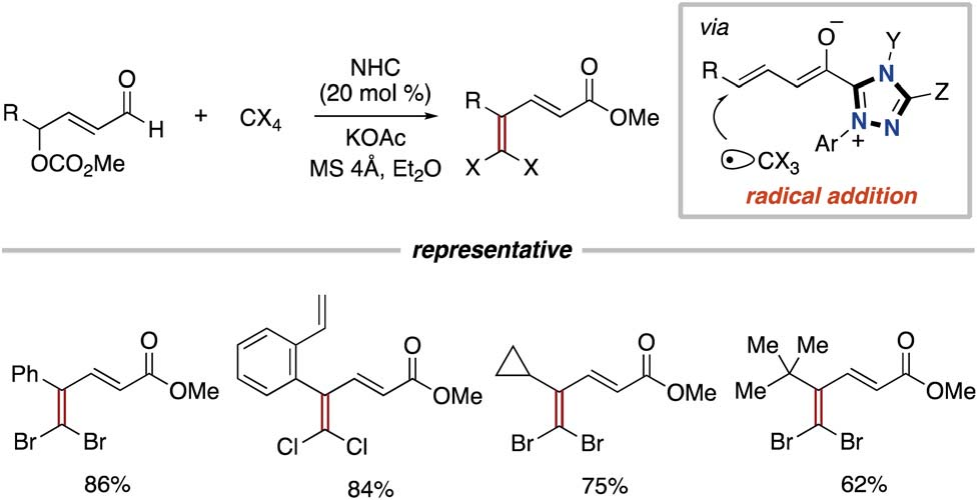
γ-Dihalomethylenation of enals by NHC and polyhalomethanes.

In addition to the use of TEMPO or nitroarenes as the SET oxidant shown above, the Sun group and the Chi group independently enabled the use of polyhalides as oxidants for the SET process. In 2016, the Sun group reported dihalomethylenation of enals. The process involves the radical addition between carbon-centered trihalomethyl radical and dienolate intermediate derived from enal and triazolium type NHC catalyst to form carbon–carbon bond formation (Scheme 6).^14^ In 2017, the Chi group used polyhalides such as CCl_4_ and C2Cl6 as oxidants in the NHC-catalyzed functionalization of aldehydes or enals.^15^ The dienolate intermediate undergoes two SET processes with polyhalides to produce the corresponding acyl triazolium intermediate.

**Scheme 7:**
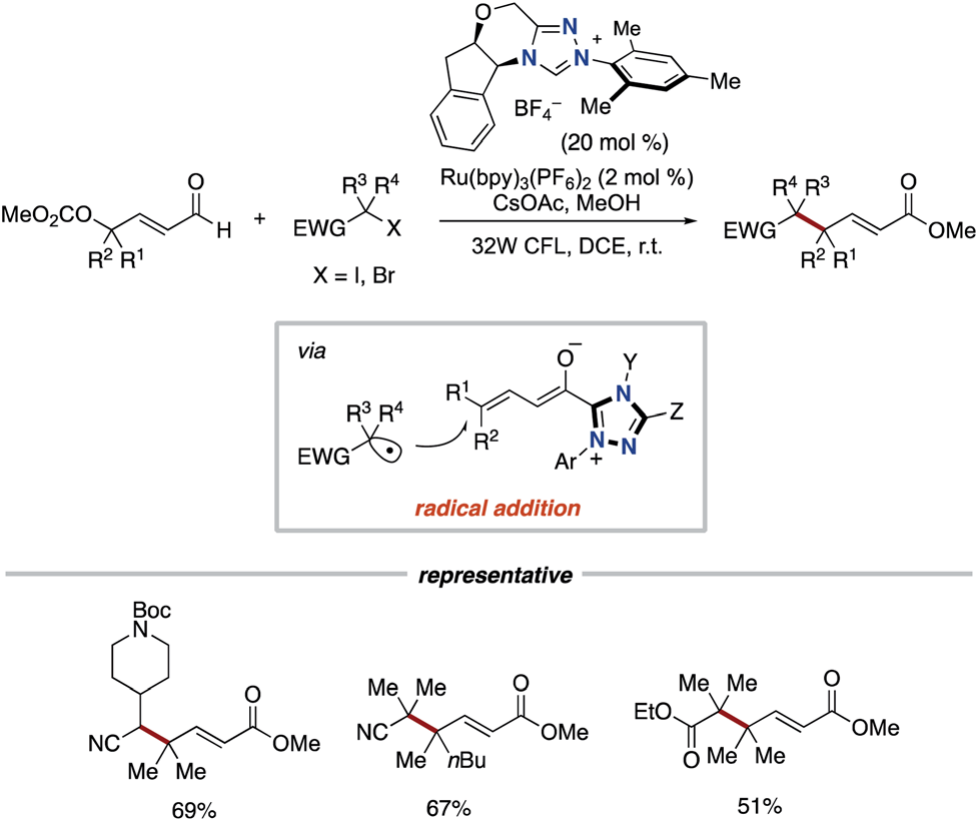
γ-Alkylation of enals with alkyl halides through synergistic Ru photoredox/NHC catalysis.

In 2019, the Ye group reported the synergistic combination of NHC catalysis with Ru photoredox catalysis for the γ- and ε-alkylation of enals with alkyl halides bearing an electron-withdrawing group such as cyano or ester to produce γ-multisubstituted-α,β-unsaturated esters (Scheme 7).^16^ The radical addition of alkyl radical, which is generated from alkyl halide by Ru photocatalysis, to dienolate intermediate derived from enal by NHC catalysis produces homoenolate radical. The subsequent SET event between the homoenolate radical and a radical cation form of Ru photocatalyst affords an acyl azolium intermediate.

**Scheme 8:**
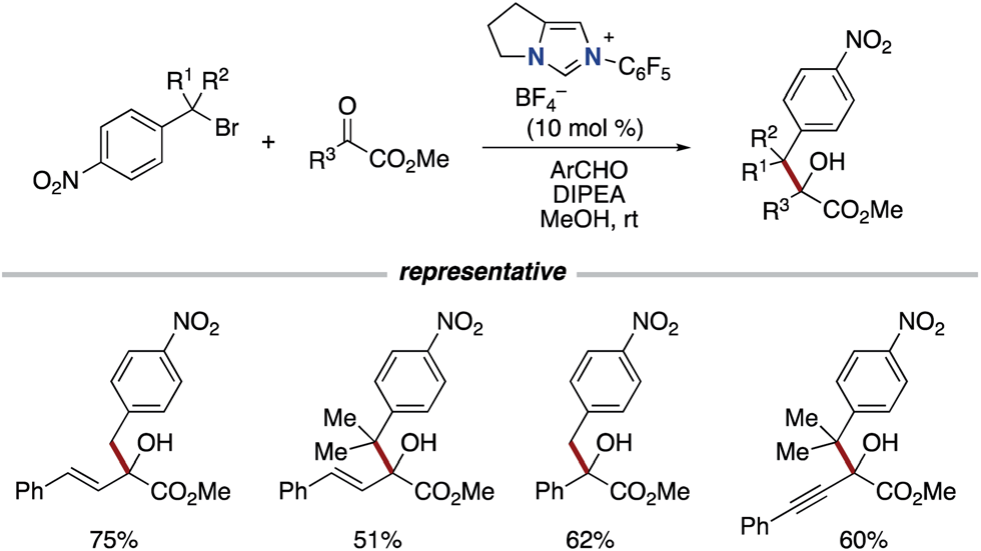
Reductive carbonyl coupling of nitrobenzyl bromides.

In 2016, the Chi group reported the reductive coupling of nitrobenzyl bromides and activated ketones or imines using aldehydes as a formal reductant (Scheme 8).^17^ The reaction involves the generation of nitrobenzyl radical intermediates from nitrobenzyl bromides followed by formal 1,2-addition. The authors did not rule out the reaction pathway involving nitrobenzyl anion generated from additional single electron reduction of the nitrobenzyl radical.

## 3. Carbon–carbon bond formation through SET/radical–radical coupling

The NHC-catalyzed radical reactions described in Chapter 2 afforded the formal oxidized product through single electron oxidations of Breslow intermediate. On the other hand, the direct radical–radical coupling of the persistent Breslow intermediate-derived radical with carbon-centered radical species has not been disclosed until quite recently.

**Scheme 9:**
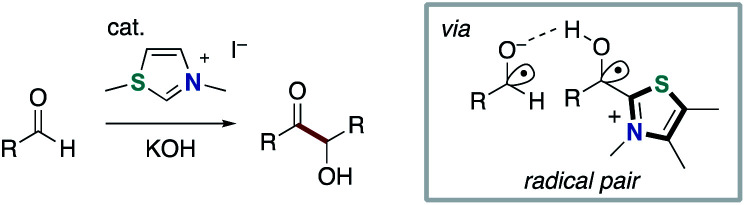
Re-evaluation of the mechanism of NHC-catalyzed Benzoin condensation.

**Scheme 10:**
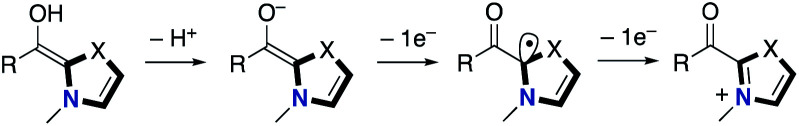
Radical intermediates in oxidative NHC catalysis.

In 2015, the Rehbein group re-evaluated the mechanism of NHC-catalyzed intermolecular Benzoin condensation (Scheme 9).^18^ Thus, radical pairs that is derived from Breslow-intermediate through SET process was observed and characterized. In 2019, Bertrand and Martin elucidated the characteristics of Breslow intermediate-derived radical species by EPR spectra, DFT calculation and CV spectrometry (Scheme 10).^19^ Then, they implied the intermediacy of captodatively stabilized radicals in NHC-catalyzed oxidative functionalization of aldehydes or enals using mild oxidants. These reports by Rehbein and Bertrand hinted that Breslow intermediate-derived radical could participate in radical–radical coupling for the bond forming reaction.

**Scheme 11:**
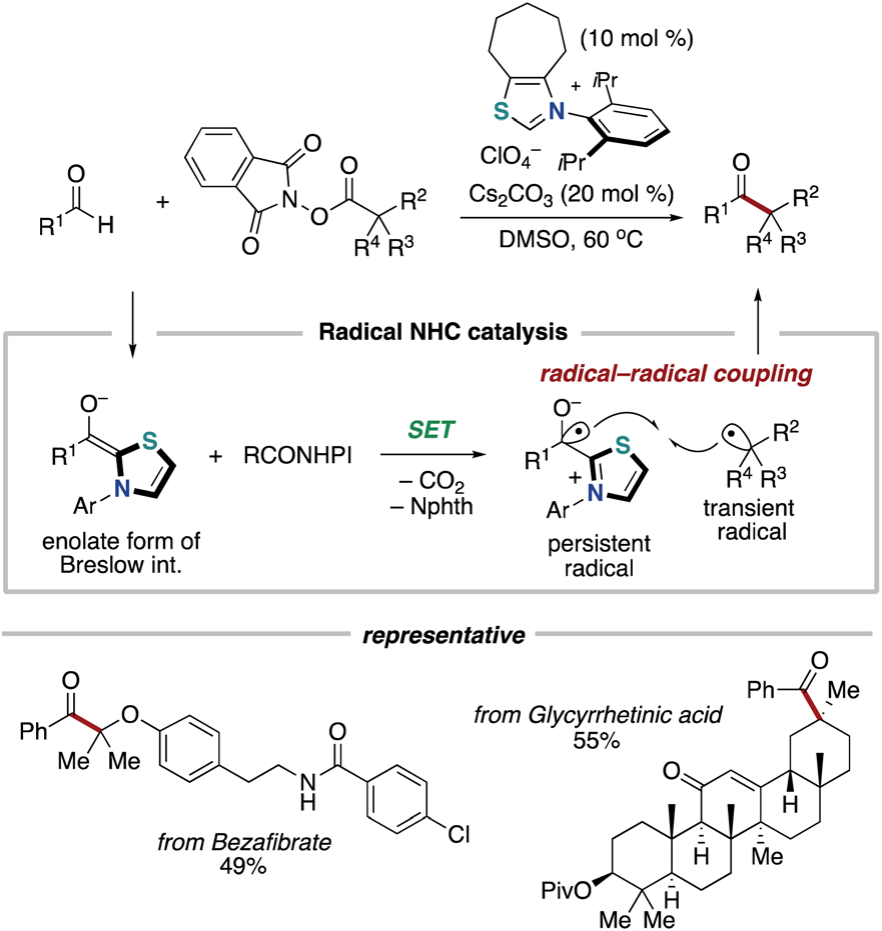
NHC-catalyzed decarboxylative alkylation of aldehydes.

In 2019, our group discovered that the persistent Breslow intermediate-derived radical couples with transient alkyl radical in the carbon–carbon bond forming process (Scheme 11).^20,21^ This unprecedented radical–radical coupling is based on kinetic phenomenon, persistent radical effect (PRE).^22^ This achievement could definitely expand the scope of the NHC-based radical catalysis. Specifically, thiazolium NHC catalysis promoted the unprecedented decarboxylative coupling of aryl aldehydes and tertiary or secondary alkyl carboxylic acid-derived redox-active esters to produce aryl alkyl ketones. The redox ester is a source of alkyl radical specie. In this report, Breslow intermediate is presumed to have necessity of deprotonation by Cs_2_CO_3_ base so that to reach enough reduction potential for SET with redox ester.^6^ Thus, an enolate form of Breslow intermediate performs SET to a redox ester, and the obtained Breslow intermediate-derived radical couples with an alkyl radical. The protocol has wide substrate scope and enabled the functionalization of pharmaceutical drugs and natural products.

**Scheme 12:**
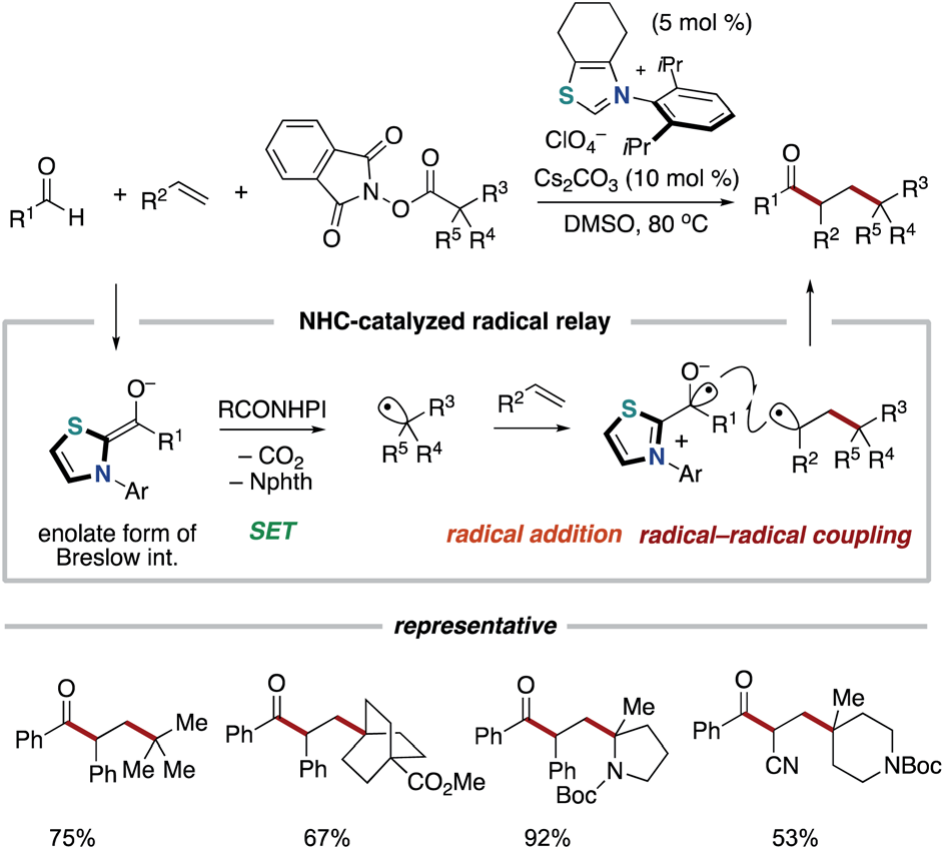
Acylalkylation of alkenes through NHC-catalyzed radical relay.

**Scheme 13:**
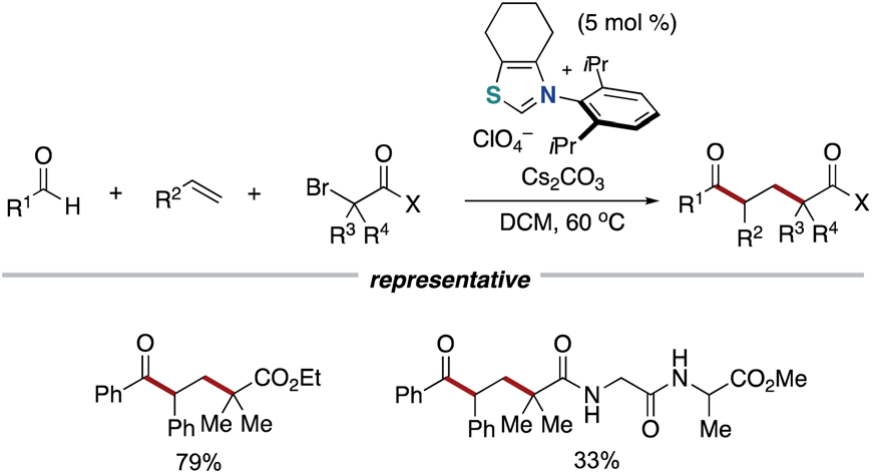
Synthesis of δ-ketocarbonyls through NHC-catalyzed radical relay.

Our group applied the NHC-based radical catalysis into three-component coupling using aldehydes, alkenes and tertiary alkyl carboxylic acid-derived redox-active esters (Scheme 12).^23^ The introduction of tertiary alkyl group and acyl group to carbon–carbon double bonds occurred with complete regioselectivity. The radical relay process involves SET from the enolate form of Breslow intermediate to a redox active ester and radical addition of the resultant alkyl radical to an alkene followed by radical–radical coupling. The key design of this radical relay process is the proper match on reaction rates of several competing radical reactions. Recently, this protocol was applied to the synthesis of δ-ketocarbonyls using tertiary α-bromocarbonyls instead of redox active esters (Scheme 13).^24^

**Scheme 14:**
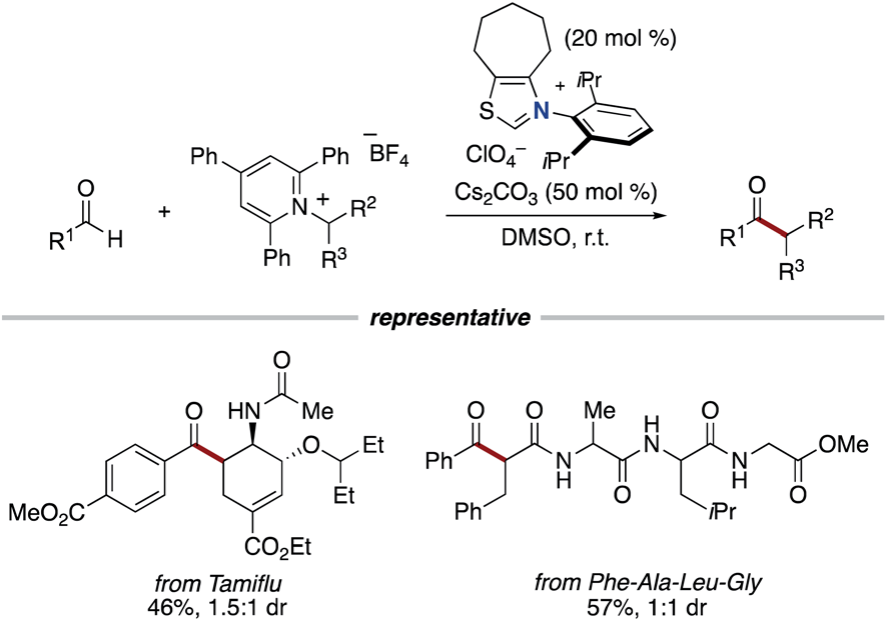
NHC-catalyzed deaminative alkylation of aldehydes.

In 2020, the Hong group enabled the use of redox active Katritzky pyridinium salts instead of redox active esters in the alkylation of aldehyde and the three-component alkylacylation of alkene (Scheme 14).^25^ Katritzky salts can be directly reduced by the enolate form of Breslow intermediate to generate a ketyl radical and an alkyl radical and then radical–radical coupling with two radical species proceeds to yield a ketone.

**Scheme 15:**
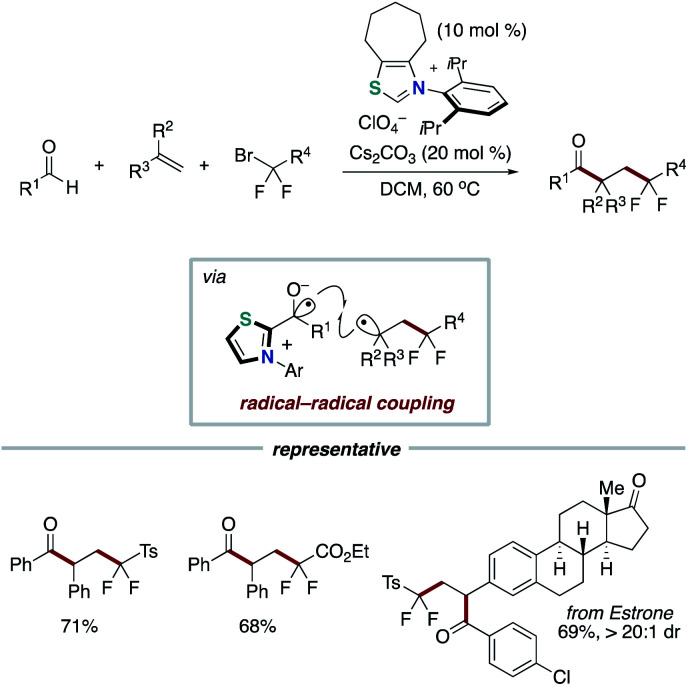
NHC-catalyzed acylfluoroalkylation of alkenes.

**Scheme 16:**
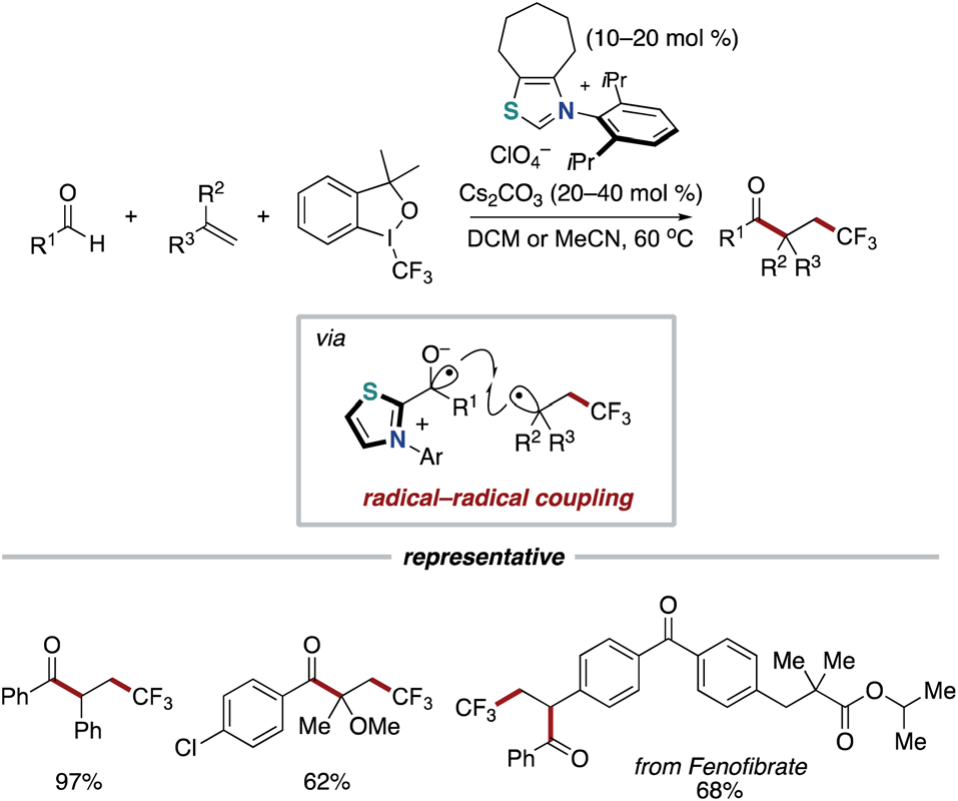
NHC-catalyzed acyltrifluorometylation of alkenes.

In 2019 and 2020, the Li group,^26^ the Wang group,^27^ and the Yang and Wu group^28^ reported the acylfluoroalkylation of alkenes based on our NHC-catalyzed three-component coupling reaction, respectively (Schemes 15 and 16). Togni I reagent or perfluoroalkylbromide instead of redox ester is used as an electron acceptor in the step of SET.

**Scheme 17:**
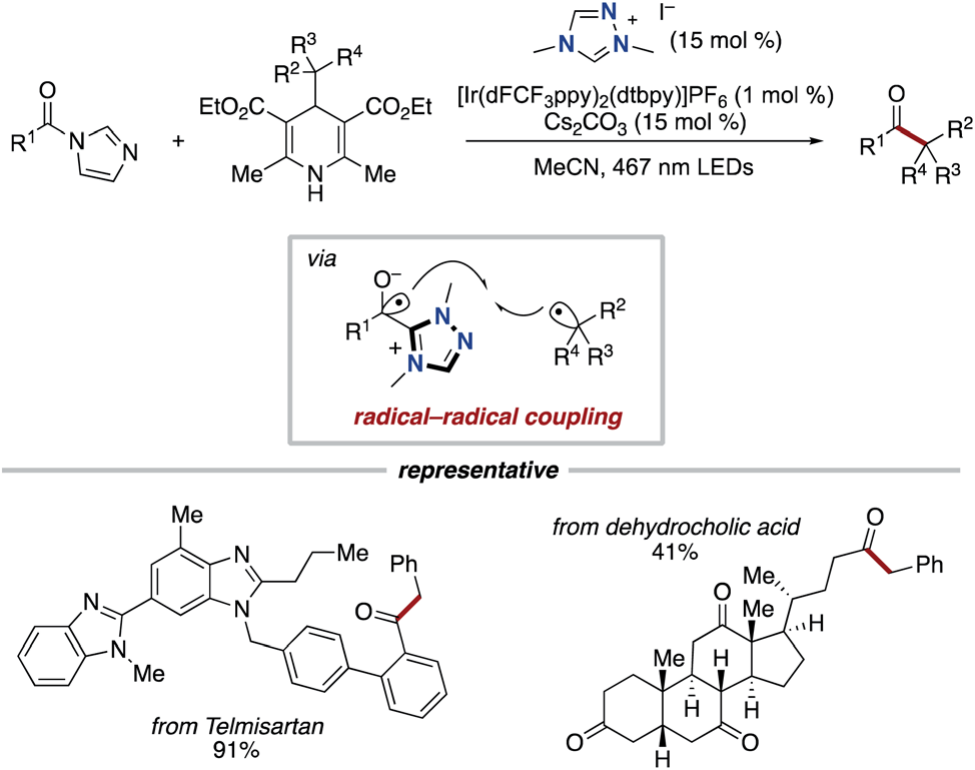
Conversion of carboxylic acids to ketones through synergistic Ir photoredox/NHC catalysis.

In 2020, the Scheidt group developed the synergistic merger of NHC-based radical catalysis and Ir photoredox catalysis for benzylation of acyl azoliums, which are prepared from carboxylic acids, to form ketones (Scheme 17).^29^ In this case, benzyl Hantzsch esters and acyl imidazoliums were used as precursor of alkyl radical and acyl azolium, respectively. The combination of NHC and Ir photoredox catalysis enabled a SET with the acyl azolium, and the subsequent radical–radical coupling with an alkyl radical allowed for the construction of a carbon–carbon bond to furnish a ketone.

**Scheme 18:**
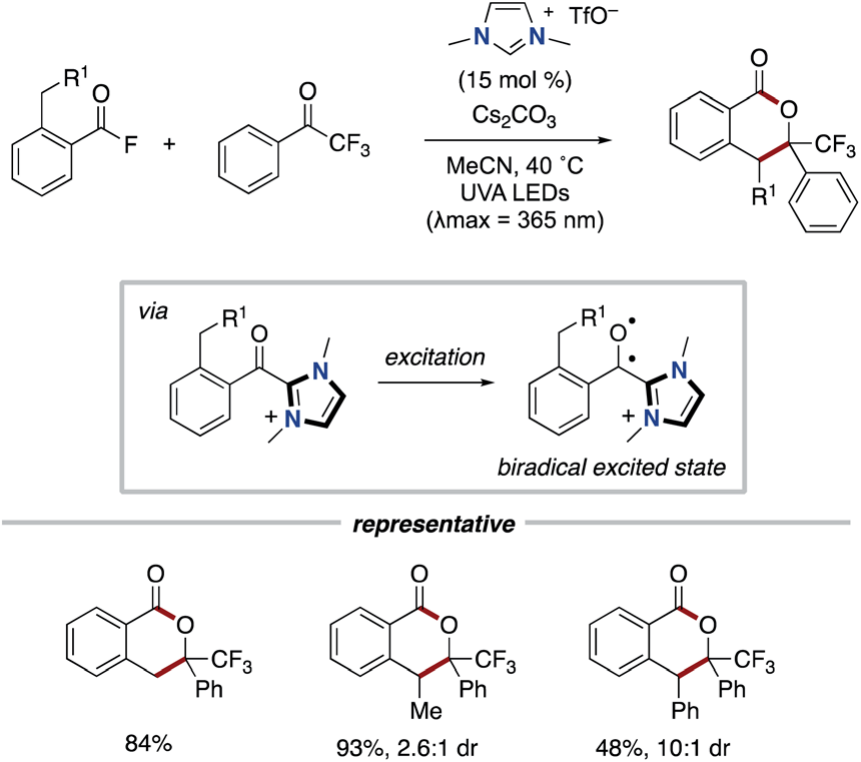
Photoenolization/Diels–Alder reaction of acid fluorides.

In 2020, the Hopkinson group reported a different type of NHC-based radical catalysis. The synergistic cooperation of NHC catalysis and light activation enables the annulation using *o*-toluoylfluorides and trifluoroacetophenones (Scheme 18).^30^ The mechanistic studies and time-dependent DFT calculations suggested that the direct excitation of acyl azolium, which is derived from acyl fluoride and NHC, by UV irradiation could create biradical-ketone-like photochemical reactivity. The triplet excited state is applied to the photoenolization and the subsequent Diels–Alder process to produce isochroman-1-one derivatives. This process does not involve the radical–radical coupling.

## 4. Conclusions

In this minireview, we summarized the NHC-catalyzed radical reaction with two categories; oxidation type reaction (Chapter 2) and carbon–carbon bond formation through SET/radical–radical coupling (Chapter 3). In particular, the finding that the persistent Breslow intermediate-derived radical couples with transient alkyl radical in the carbon–carbon bond forming process described in Chapter 3, opened the door to a new design guideline for NHC organocatalysis. Although these NHC-catalyzed radical reactions enabled the solution of various problems in organic synthesis, significant future contributions will be needed in terms of the development of stereoselective and universal bond-formation reactions, the application to biomolecules and the mechanistic studies by intermediate analysis and theoretical calculations.

## Conflicts of interest

There are no conflicts to declare.
